# Ingestion of single guide RNAs induces gene overexpression and extends lifespan in *Caenorhabditis elegans via* CRISPR activation

**DOI:** 10.1016/j.jbc.2022.102085

**Published:** 2022-05-27

**Authors:** Fabian Fischer, Christoph Benner, Anita Goyala, Giovanna Grigolon, Davide Vitiello, JiaYee Wu, Kim Zarse, Collin Y. Ewald, Michael Ristow

**Affiliations:** 1 Energy Metabolism Laboratory, Department of Health Sciences and Technology, Institute of Translational Medicine, Swiss Federal Institute of Technology (ETH) Zurich, Schwerzenbach, Switzerland; 2 Science and Policy Program, Life Science Zurich Graduate School, Zurich, Switzerland; 3 Extracellular Matrix Regeneration Laboratory, Department of Health Sciences and Technology, Institute of Translational Medicine, Swiss Federal Institute of Technology (ETH) Zurich, Schwerzenbach, Switzerland; 4 Charité – Universitätsmedizin Berlin, Freie Universität Berlin and Humboldt-Universität zu Berlin, Institute of Experimental Endocrinology and Diabetology, Berlin, Germany

**Keywords:** aging, *C. elegans*, CRISPR/Cas9, gene expression, synthetic biology, HIF-1, HSF-1, cDNA, complementary DNA, CGC, Caenorhabditis Genetics Center, CRISPRa, CRISPR activation, FDR, false discovery rate, HA, hemagglutinin, NGM, nematode growth medium, sgRNA, single-guide RNA, TSS, transcription start site

## Abstract

Inhibition of gene expression in *Caenorhabditis elegans*, a versatile model organism for studying the genetics of development and aging, is achievable by feeding nematodes with bacteria expressing specific dsRNAs. Overexpression of hypoxia-inducible factor 1 (*hif-1*) or heat-shock factor 1 (*hsf-1*) by conventional transgenesis has previously been shown to promote nematodal longevity. However, it is unclear whether other methods of gene overexpression are feasible, particularly with the advent of CRISPR-based techniques. Here, we show that feeding *C. elegans* engineered to stably express a Cas9-derived synthetic transcription factor with bacteria expressing promoter-specific single guide RNAs (sgRNAs) also allows activation of gene expression. We demonstrate that CRISPR activation *via* ingested sgRNAs specific for the respective promoter regions of *hif-1* or *hsf-1* increases gene expression and extends lifespan of *C. elegans*. Furthermore, and as an *in silico* resource for future studies aiming to use CRISPR activation in *C. elegans*, we provide predicted promoter-specific sgRNA target sequences for >13,000 *C. elegans* genes with experimentally defined transcription start sites. We anticipate that the approach and components described herein will help to facilitate genome-wide gene overexpression studies, for example, to identify modulators of aging or other phenotypes of interest, by enabling induction of transcription by feeding of sgRNA-expressing bacteria to nematodes.

Targeted inhibition of gene expression by RNAi with transcript-specific dsRNAs has greatly facilitated the systematic analyses of genetic pathways in eukaryotic organisms (1, 2, 3, 4). Because of its widespread research impact, the seminal discovery of dsRNA-mediated RNAi by Fire *et al.*, was awarded with the Nobel Prize in Physiology or Medicine in 2006. In the nematode *Caenorhabditis elegans*, RNAi-mediated knockdown of specific transcripts can be conveniently achieved by feeding nematodes with bacteria expressing appropriate dsRNAs (3, 5, 6). This approach continues to be an essential tool for *C. elegans* research (7) and has enabled several genome-wide knockdown screens (1, 3, 8). Currently, no complementary method for overexpression of *C. elegans* genes with similar ease and flexibility exists.

The CRISPR-Cas system was initially described as a type of bacterial adaptive immune defense, able to protect prokaryotic organisms against viral or plasmid infections (9, 10, 11, 12). Soon after, it was utilized for the purpose of genome editing, mainly by introducing the Cas9 protein from *Streptococcus pyogenes* into other organisms (13, 14, 15, 16, 17). The versatility of Cas9 is largely based on its ability to be directed by a single-guide RNA (sgRNA) toward a desired DNA sequence (14). WT Cas9 introduces DNA double-strand breaks at the targeted location and is thus an RNA-guided DNA endonuclease. In this capacity, Cas9 has been used in several models, such as *C. elegans* (18), *Drosophila melanogaster* (19), mice (20), and human cells (21), for targeted gene deletions and to introduce specific sequence modifications. The Cas9 protein has since been adapted for various other molecular biology applications (22, 23, 24). Through mutation of its two core catalytic residues to alanine, Cas9 is rendered fully inactive as an endonuclease. The resulting inert RNA-guided DNA-binding protein, called nuclease–dead Cas9 (dCas9), can then be modularly fused with different functional domains. Examples include its fusion with transactivation (25, 26) or DNA-methylase domains (27), turning dCas9 into an RNA-guided transcription factor or RNA-guided DNA-modifying enzyme, respectively.

Overexpression of genes by utilizing dCas9 fused with a transactivation domain (dCas9^TA^) and promoter-specific sgRNAs, here and by others termed CRISPR activation or CRISPRa for short (28), has been demonstrated to be feasible in, for example, *D*. *melanogaster* (29), zebrafish (30), and different human tissue culture models (25, 26, 31, 32). The use of CRISPRa has also been previously explored in at least two independent *C. elegans* studies (30, 33). In both cases, CRISPRa was implemented using a technically rather demanding delivery of necessary components to nematodes by microinjection, preventing its use for large-scale or genome-wide screening purposes. To our knowledge, only one study so far examined the possibility of feeding sgRNA-expressing bacteria to *C. elegans*, specifically in the context of classical Cas9-mediated genome editing (34). This approach demonstrated that bacterial delivery of sgRNAs to nematodes is possible in general (34) but did not evaluate its feasibility for targeted gene overexpression and was found to be of limited utility (35).

Here, we combine the aforementioned approaches to establish a novel method for inducing transcription of endogenous genes in *C. elegans* by (a) generating and validating a *C. elegans* strain stably harboring an expression-optimized variant of dCas9 fused with the well-characterized VP64 transactivation domain (25, 26, 32, 36) and (b) establishing a variant of the L4440 RNAi vector containing a scaffold for expression of *C. elegans* promoter-specific sgRNAs in *Escherichia coli*. By combining these components, focusing on two genes previously linked to the control of aging phenotypes (*hif-1* (37) and *hsf-1* (38)) for proof-of-principle purposes, we show that they are sufficient to achieve gene overexpression in *C. elegans* by feeding of sgRNA-expressing bacteria. Furthermore, we demonstrate that known *C. elegans* longevity phenotypes associated with increased expression of *hif-1* and *hsf-1*, respectively, by conventional methods can be achieved by our method relying on ingested sgRNAs. We furthermore provide an *in silico* library of *C. elegans* promoter-specific sgRNA target sequences, covering the promoters of more than 13,000 *C. elegans* genes with experimentally defined representative transcription start sites (TSSs) as previously identified (39). Thus, feeding-based CRISPRa in *C. elegans* is demonstrated as an alternative and comparatively simple method for gene overexpression, similar in concept to feeding-based RNAi for gene inactivation purposes.

## Results

**Figure 1:**
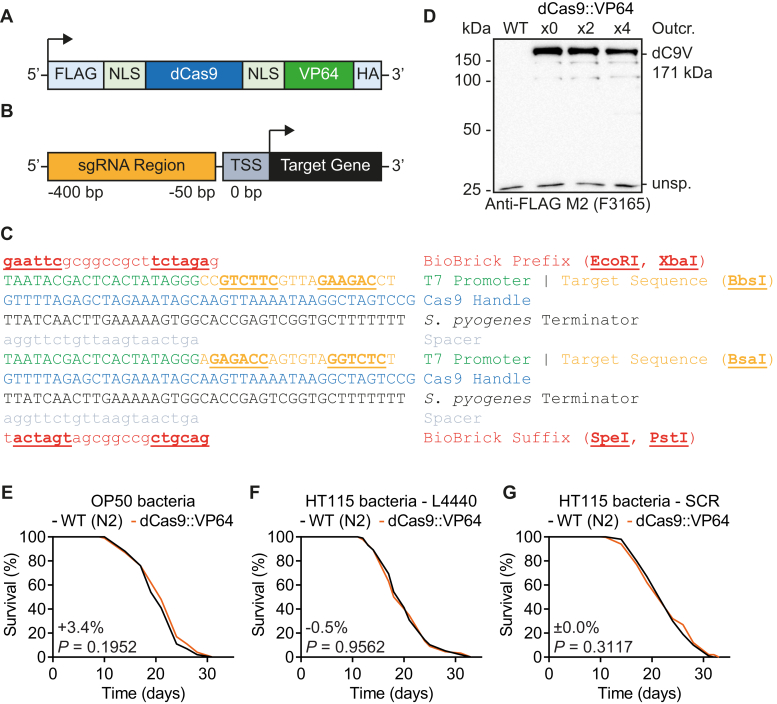
**Components for CRISPR activation in *C. elegans* by bacterial delivery of sgRNAs.***A*, domain organization of the dCas9::VP64 fusion protein, including two nuclear localization signals (NLSs), and a FLAG- and HA-tag. *B*, schematic representation of the region upstream of a given transcription start site (TSS) from which promoter-specific sgRNAs are selected. *C*, full sequence of the two sgRNA expression cassettes in vector L4440_BioBrick-sgRNA, including BioBrick cloning sites and with individual features as indicated by color. *D*, Western blot analysis of total protein extracts from WT N2 nematodes *versus* dCas9::VP64 nematodes outcrossed x0, x2, or x4 (MIR249). The 171 kDa dCas9::VP64 protein (dC9V) is detected with a FLAG antibody in all dCas9::VP64 nematodes and an additional unspecific (unsp.) band is detected in all samples at 25 kDa. *E*–*G*, lifespan assay of WT *versus* dCas9::VP64 nematodes on OP50 bacteria (*E*), HT115 bacteria carrying the L4440 vector (*F*), or HT115 sgRNA scramble control (SCR) bacteria (*G*). *p*-values of *C. elegans* lifespan assays were determined by log-rank test. See Table S1 for detailed lifespan assay statistics. HA, hemagglutinin; sgRNA, single-guide RNA.

To allow dCas9^TA^-mediated overexpression of genes in *C. elegans* by bacterially delivered promoter-specific sgRNAs, a vector encoding a *C. elegans* expression-optimized dCas9 fused with the VP64 transactivation domain (dCas9::VP64; Fig. 1*A*), controlled by the ubiquitous *sur-5*/K03A1.5 (WormBase WBGene00006351) promoter, was constructed and stably introduced into nematodes by biolistic bombardment. It has been previously established that the *sur-5* gene is expressed across virtually all *C. elegans* tissues and stages of the nematodal life cycle (40). Accordingly, the *sur-5* promoter is hence frequently used for ubiquitous and constitutive *C. elegans* transgenic overexpression purposes (41, 42, 43).

Promoter-specific sgRNA target sequences were selected from regions −50 to −400 bp upstream of the respective TSS (Fig. 1*B*), following previously established sgRNA design rules (44). Note that the designation of TSSs in *C. elegans* is hampered by the frequently occurring phenomenon of *trans*-splicing, that is, the replacement of the 5′ UTR of a pre-mRNA transcript with a short common RNA sequence called the spliced leader. *Trans*-splicing in *C. elegans* has been estimated to affect up to 70% of mRNAs, which masks their original 5′ UTR and thereby impedes mapping of TSSs and relevant promoter regions (39, 45). As detailed in the experimental procedures, this phenomenon was explicitly taken into account when selecting *C. elegans* promoter-specific sgRNAs. For the expression of *C. elegans* promoter-specific sgRNAs in *E*. coli HT115 bacteria, the L4440 RNAi empty vector (Addgene; plasmid #1654) was modified to contain two consecutive sgRNA expression cassettes (14, 46) under control of individual T7 promoters and together flanked by BioBrick cloning sites (47) (Fig. 1*C*), resulting in the vector L4440_BioBrick-sgRNA.

First, expression of the 171 kDa dCas9::VP64 fusion protein was tested for by immunoblotting, indicating that it was present in initially bombarded dCas9::VP64 nematodes and still retained after two and four rounds of outcrossing against WT N2 nematodes (Fig. 1*D*). In addition, expression of the dCas9::VP64 protein, which contains a FLAG-tag and hemagglutinin (HA)-tag, was confirmed by immunofluorescence microscopy in outcrossed dCas9::VP64 nematodes using an anti-HA antibody (Fig. S1*A*).

For unknown reasons, nonoutcrossed dCas9::VP64 nematodes showed a reduced lifespan *versus* the WT control on OP50 bacteria (mean −13.0%, *p*-value < 0.0001; Fig. S1*B*), while the lifespans of strains outcrossed twice (Fig. S1*C*) or four times (Fig. 1*E*) were not different from WT. The four-times outcrossed dCas9::VP64 strain was used for all further experiments, and additionally confirmed to not display a lifespan phenotype when raised on HT115 bacteria containing the L4440 RNAi empty vector (HT115 L4440; Fig. 1*F*). Next, HT115 bacteria expressing scramble control (SCR) sequences from L4440_BioBrick-sgRNA, using two different sgRNA SCR vectors A and B, were tested for their influence on *C. elegans* aging when compared to HT115 L4440. Lifespans of both WT and dCas9::VP64 nematodes remained unaltered on either of the HT115 SCR bacteria compared to HT115 L4440 (Fig. S1, *D*–*G*). Thus, HT115 SCR A and B bacteria, together hereafter referred to as HT115 SCR, were used interchangeably as a control for all subsequent experiments. The lifespan of dCas9::VP64 nematodes *versus* WT raised on HT115 SCR was assayed and, again, no appreciable difference of lifespans was observed (Fig. 1*G*). Together, these results demonstrate that neither presence of the dCas9::VP64 protein in the outcrossed strain nor feeding with HT115 SCR bacteria has any discernible impact on *C. elegans* lifespan (see Table S1 for detailed statistics of lifespan assays and repeats thereof performed throughout this study). Furthermore, overall transcriptomic changes of dCas9::VP64 strain *versus* WT, raised on HT115 SCR bacteria, were assessed by RNA-Seq (full data available in NCBI's Gene Expression Omnibus, GEO Series accession number GSE202213 [https://www.ncbi.nlm.nih.gov/geo/query/acc.cgi?acc=GSE202213]). Thereby, only 94 of 12,824 transcripts with feature counts above threshold were detected as significantly differentially regulated between the two strains. Notably, transcription of the genes used for proof of principle in this study was not at all detectably affected (*hif-1* fold-change dCas9::VP64 *versus* WT = 0.954, *p* = 0.350, false discovery rate (FDR) = 0.627; *hsf-1* fold-change dCas9::VP64 *versus* WT = 1.049, *p* = 0.303, FDR = 0.584).

Next, promoter-specific sgRNA target sequences for the promoters of the genes *hif-1*/F38A6.3 (WBGene00001851) and *hsf-1*/Y53C10A.12 (WBGene00002004), four each in total, were selected from the appropriate region (*i.e.*, −50 to −400 bp upstream of the respective TSS) and inserted in pairs into L4440_BioBrick-sgRNA. This resulted in two sgRNA expression vectors for each gene, referred to in short as sgRNA *hif-1* A and B, as well as sgRNA *hsf-1* A and B, respectively (Table S2). Each vector was individually transformed into HT115 bacteria.

**Figure 2:**
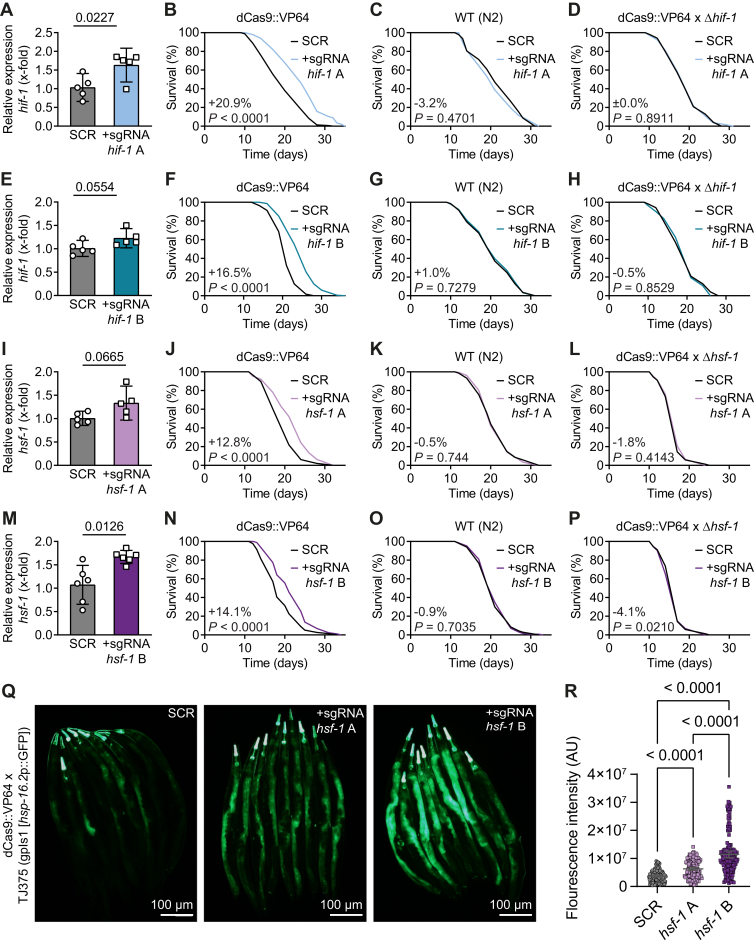
**Increased gene expression and lifespan by CRISPRa with ingested sgRNAs.***A*, relative *hif-1* expression in dCas9::VP64 nematodes fed with HT115 sgRNA scramble control (SCR) or HT115 sgRNA *hif-1* A bacteria, as determined by RT-qPCR. *B*, lifespan assay with nematodes and bacteria as in (*A*). *C*, lifespan assay with bacteria as in (*A*) and using WT N2 nematodes. *D*, lifespan assay with bacteria as in (*A*) and using dCas9::VP64 nematodes with a simultaneous *hif-1* loss-of-function mutation (dCas9::VP64xΔ*hif-1*, MIR250). *E*–*H*, similar experiments as in (*A*–*D*), using HT115 sgRNA *hif-1* B bacteria instead. *I*–*L*, similar experiments as in (*A*–*D*), using HT115 sgRNA *hsf-1* A bacteria and dCas9::VP64xΔ*hsf-1* (MIR251). *M*–*P*, similar experiments as in (*A*–*D*), using HT115 sgRNA *hsf-1* B bacteria and dCas9::VP64xΔ*hsf-1*. *Q*, representative images showing expression of *hsp-16.2* promoter-driven GFP in dCas9::VP64 x TJ375 (gpIs1 [*hsp-16.2*p::GFP]) (MIR276) animals fed with HT115 SCR or HT115 sgRNA *hsf-1* A or B bacteria. *R*, scatter plots (mean and 95% CI) showing quantification data for *hsp-16.2*p::GFP expression in animals as in (*Q*) from four independent replicate experiments (SCR *n* = 145, sgRNA *hsf-1* A *n* = 135, sgRNA *hsf-1* B *n* = 147 animals). Data in bar graphs are mean ± SEM, with individual data points representing biological replicates and *p*-values determined with two-tailed unequal variances *t* tests. *p*-values of *C. elegans* lifespan assays were determined by log-rank test. See Table S1 for detailed lifespan assay statistics. CRISPRa, CRISPR activation; RT-qPCR, reverse transcription-quantitative PCR; sgRNA, single-guide RNA.

Feeding dCas9::VP64 nematodes with HT115 bacteria containing the sgRNA *hif-1* A vector increased *hif-1* expression mildly but significantly (1.63 ± 0.16 SEM, *p* = 0.0227), as determined by quantitative PCR *versus* feeding with HT115 SCR (Fig. 2*A*). More strikingly, both mean and maximum lifespan of dCas::VP64 nematodes fed with HT115 sgRNA *hif-1* A were extended *versus* the control (mean +20.9%, 75% max 28 *versus* 24 days, *p* < 0.0001; Fig. 2*B*), and this overall effect was confirmed in three independent lifespan assays (Table S1). Notably, this increase in lifespan following sgRNA-mediated *hif-1* overexpression was comparable to what was previously observed with transgenic strains with stable integrations of *hif-1*p::*hif-1*::*myc* overexpression constructs, generated by classical biolistic bombardment (37), and also to our own lifespan assays using this transgenic strain (ZG580), independent of the bacterial food source (OP50, HT115 L4440, or HT115 SCR; Fig. S1, *H*, *J* and *L* and Table S1). To test the specificity of this effect on lifespan, feeding of HT115 sgRNA *hif-1* A to either WT nematodes or dCas9::VP64 nematodes carrying a homozygous *hif-1* loss-of-function allele (dCas9::VP64xΔ*hif-1*) was assayed. In the absence of the dCas9::VP64 fusion protein (Fig. 2*C*), or functional *hif-1* (Fig. 2*D*), feeding with HT115 sgRNA *hif-1* (A) did not affect lifespan. Performing the same set of experiments as in 2*A*–2*D* with HT115 sgRNA *hif-1* B yielded very similar results (Fig. 2, *E*–*H*). While *hif-1* expression following feeding with HT115 sgRNA *hif-1* B was increased only by trend (1.23 ± 0.07 SEM, *p* = 0.0554; Fig. 2*E*), again a clear effect on the mean and maximum lifespan of dCas9::VP64 nematodes was detected (mean +16.5%, 75% max 28 *versus* 23 days, *p* < 0.0001; Fig. 2*F*) and found to be overall reproducible in independent experiments (Table S1). This increase in lifespan following feeding with HT115 sgRNA *hif-1* B also required the presence of the dCas9::VP64 fusion protein (Fig. 2*G*) and functional *hif-1* (Fig. 2*H*).

Feeding dCas9::VP64 nematodes with HT115 bacteria containing the sgRNA *hsf-1* A or B vector in both cases, by trend or significantly, increased *hsf-1* expression (A: 1.33 ± 0.13 SEM, *p* = 0.0665; Fig. 2*I* | B: 1.67 ± 0.05 SEM, *p* = 0.0126; Fig. 2*M*) and mean and maximum lifespan (A: mean +12.8%, 75% max 24 *versus* 21 days, *p* < 0.0001; Fig. 2*J* | B: mean +14.1%, 75% max 25 *versus* 21 days, *p* < 0.0001; Fig. 2*N*) in a reproducible manner (Table S1). These observations were again congruent with published data on lifespan extension of a transgenic *hsf-1* overexpressor (38) and our own lifespan assays with this very strain (CF1824; Fig. S1, *I*, *K* and *M*, and Table S1). Similar as observed for *hif-1*, HT115 sgRNA *hsf-1* A and B were unable to extend lifespan when applied to either WT nematodes lacking the dCas9::VP64 fusion protein (Fig. 2, *K* and *O*) or to dCas9::VP64 nematodes with a simultaneous loss-of-function mutation of *hsf-1* (dCas9::VP64xΔ*hsf*-1; Fig. 2, *L* and *P*). Feeding of sgRNA *hif-1* or *hsf-1* A and B bacteria to the respective transgenic overexpression strain for *hif-1* (ZG580) or *hsf-1* (CF1824) also did not significantly affect lifespan of these strains compared to feeding with HT115 SCR (Fig. S1, *N*–*Q* and Table S1), again as to be expected in absence of the dCas9::VP64 protein.

As an additional phenotypic readout for *hsf-1* overexpression, we quantified *hsp-16.2* promoter-driven GFP expression in a newly generated dCas9::VP64 x TJ375 (gpIs1 [*hsp-16.2*p::GFP]) reporter strain, with *hsp-16.2* being a well-described downstream target gene of the HSF-1 transcription factor (48). Feeding this strain with HT115 sgRNA *hsf-1* A or B bacteria in both cases led to a significant increase of the detectable GFP signal over feeding with HT115 SCR bacteria, with feeding of sgRNA *hsf-1* B bacteria having a stronger effect (Fig. 2, *Q* and *R*).

Finally, also as a resource for future studies in the *C. elegans* scientific community, we computationally predicted promoter-specific sgRNA target sequences, applying the same design rules that were followed to select the *hif-1* and *hsf-1* promoter-specific sgRNA target sequences used in the proof-of-principle experiments presented herein. Taking into account the phenomenon of *trans*-splicing and the *C. elegans* TSS landscape as mapped by Saito *et al.* (39), we generated a library of 20 nucleotide *C. elegans* promoter-specific sgRNA target sequences, located −50 to −400 bp upstream of the respective embryonic and/or adult TSS, for more than 13,000 genes (Table S3) (please refer to the Experimental procedures for further details). Notably, the thus predicted sgRNA target sequences for *hif-1* and *hsf-1* were confirmed to contain those that were selected manually and used in vectors sgRNA *hif-1* A and B and sgRNA *hsf-1* A and B for proof-of-principle experiments, as depicted previously.

## Discussion

We here show that the implementation of CRISPRa by ingested sgRNAs in *C. elegans* is a feasible approach to induce gene expression. Specifically, our results demonstrate that the here established components, meaning nematodes stably expressing dCas9::VP64 and bacterial vectors expressing *C. elegans* promoter-specific sgRNAs in *E. coli* HT115, are sufficient to detectably increase expression of targeted genes and to elicit additional phenotypic effects. Increases in lifespan when overexpressing *hif-1* and *hsf-1* by feeding-based CRISPRa are shown to be specific for the individual components and targeted genes and are phenotypically congruent with observations in transgenic *hif-1* and *hsf-1* overexpressing animals (37, 38). While previous studies in *C. elegans* have already shown CRISPRa to be achievable by delivery of necessary components to nematodes by microinjection (30, 33), feasibility of sgRNA delivery by bacteria for this purpose, as here demonstrated, has apparently not been explored. Notably, the degree of CRISPRa gene overexpression observed in these studies was, similar to our results, somewhat limited. This is not necessarily a problem, since low-level overexpression of a given transgene by classical methods may yield the opposite phenotype of high-level overexpression; we recently have shown that limited overexpression of Grainyhead 1 (*ghr-1*) following transgene bombardment in *C. elegans* promotes longevity, whereas high-level overexpression of the same transgene shortens lifespan (49).

Further optimization of individual components used for CRISPRa in *C. elegans*, for example, by testing additional variants of transactivation domains fused to dCas9, different promoters (with varying tissue specificity or inducibility, as opposed to the ubiquitous and constitutive *sur-5* promoter here used) driving expression of such dCas9^TA^ variants, other dCas proteins instead of dCas9, and/or different bacterial sgRNA expression cassettes, might considerably increase efficiency and lead to more pronounced and better detectable effects. Assaying efficiency of feeding-based CRISPRa on a single nematode level and in different tissues by using distinct and individually scorable readouts, especially when implementing different combinations of aforementioned variable components, might be particularly valuable for future comparative methodological studies in this regard.

Nevertheless, feeding-based CRISPRa, as established here, significantly simplifies gene overexpression compared to methods commonly employed so far. These usually require (a) several cloning steps to generate vectors in which a suitable promoter controls expression of the desired gene or transcript, (b) technically demanding delivery of such vectors to nematodes (injection or bombardment), and (c) extensive screening procedures to identify stable overexpression mutants, followed by (d) several rounds of backcrossing to avoid unspecific effects.

Given the possibility of sgRNAs bacterially delivered to *C. elegans* in directing Cas9 variants toward a desired DNA sequence, as also demonstrated elsewhere (34), additional methods following the same general concept appear quite promising. For example, a fusion of dCas9 to histone modifiers allows control of various epigenetic modifications in a defined manner (50). Introducing appropriate dCas9 variants to *C. elegans* could thus be suitable for spatially and temporally defined editing of epigenetic states by supplying appropriate sgRNA-expressing bacteria. Overall, the versatility and modularity of feeding-based dCas9 targeting in *C. elegans* offers a host of opportunities for scalable techniques of targeted genomic manipulation in this organism.

### Note added after acceptance

While this manuscript was under final review, another study that implemented CRISPRa in combination with feeding of sgRNAs in *C. elegans* was published elsewhere online ahead of print (51). Instead of using an *S*. *pyogenes* Cas9-derived synthetic transcription factor to activate *hif-1* and *hsf-1* expression, Luo *et al.* opted to use a *Camphylobacter jejuni* Cas9-derived variant and focused on a different set of exemplary target genes (including *aak-2*, *lipl-4*, and *pha-4*), otherwise using a very similar approach to ours and also showing effects both on mRNA expression and lifespan. We are very pleased to see that a team of colleagues independently found feeding of sgRNAs to be sufficient for CRISPRa in *C. elegans* and believe this only adds to further strengthen overall validity of this technique and incentivize its use in the *C. elegans* scientific community.

## Experimental procedures

### *C. elegans* strains and maintenance

The following *C. elegans* strains used for this publication were provided by the Caenorhabditis Genetics Center (CGC at the University of Minnesota): N2 (*C. elegans* wild isolate variant Bristol), HT1593 (*unc-119*(*ed3*) III.), ZG31 (*hif-1*(*ia4*) V.), PS3551 (*hsf-1*(*sy441*) I.), ZG580 (*unc-119*(*ed3*) III; iaIs28 [*hif-1p*::*hif-1a*::tag + unc-119(+)]), CF1824 (muEx265 [*hsf-1p*::*hsf-1*(cDNA) + myo-3::GFP]), and TJ375 (gpIs1 [*hsp-16.2*p::GFP]). We newly generated the strain dCas9::VP64 (K03A1.5p::3xFLAG::SV40-NLS::dCas9::SV40-NLS::VP64::HA + *unc-119*(+)) by biolistic bombardment of HT1593 with a dCas9::VP64 overexpression vector as detailed later. Unless explicitly stated otherwise, we used this dCas9::VP64 strain outcrossed four times against WT N2, the resulting outcrossed strain termed MIR249 (risIs33), for all corresponding experiments reported in this publication. For the generation of dCas9::VP64 nematodes with additional loss-of-function mutations of *hif-1* or *hsf-1*, MIR249 was intercrossed with strain ZG31 or PS3551, respectively. The resulting strains are termed MIR250 (MIR249 intercrossed with ZG31) and MIR251 (MIR249 intercrossed with PS3551). MIR249 was additionally intercrossed with strain TJ375 to generate MIR276, resulting in a strain that was used for *hsp-16.2*p::GFP fluorescence microscopy experiments. All newly generated MIR strains (249, 250, 251, and 276) have been deposited at the CGC. For maintenance, nematodes were grown on nematode growth medium (NGM) agar plates in 90 mm petri dishes at 20 °C using *E. coli* OP50 bacteria as a food source (52). NGM agar plates, after pouring, were dried at room temperature (RT) for 1 to 2 days and then stored at 4 °C until further use.

### *E. coli* strains and culturing

*E. coli* OP50 bacteria (CGC) were streaked out on DYT (16 g/l tryptone, 10 g/l yeast extract, 5 g/l NaCl, pH = 7.0 with NaOH) agar plates, and single colonies picked from such plates were cultured overnight at 37 °C and constant shaking in Erlenmeyer flasks containing liquid DYT medium. Bacterial overnight cultures were concentrated by centrifugation for 30 min at 3200*g* and 4 °C. The prepared bacteria were spotted on NGM agar plates and allowed to grow for 16 to 24 h prior to use.

*E*. *coli* HT115(DE3) bacteria (CGC), containing either the standard L4440 RNAi empty vector or one of the vectors derived from the L4440_BioBrick-sgRNA vector (see later), were streaked out on LB agar plates with 100 μg/ml ampicillin and 12.5 μg/ml tetracycline, and single colonies picked from such plates were cultured overnight at 37 °C and constant shaking in Erlenmeyer flasks containing liquid LB medium with 100 μg/ml ampicillin. Bacterial overnight cultures were concentrated by centrifugation for 30 min at 3200*g* and 4 °C. The prepared bacteria were spotted on NGM agar plates additionally containing 100 μg/ml ampicillin and 1 mM IPTG and allowed to grow for 16 to 24 h prior to use (all reagents from AppliChem).

The *E. coli* strain NEB 5-alpha (New England Biolabs, catalog no.: #C2987) was used according to the manufacturer’s instructions for all cloning procedures as described later.

### Design of *C. elegans* promoter-specific sgRNA target sequences

To predict sgRNA target sequences specific for the promoter of a particular *C. elegans* gene, stringent design rules were defined. These rules were based on known properties of the *C. elegans* TSS landscape (39) and established guidelines for the selection of maximally efficient and specific promoter-localized sgRNA target sequences (44, 53, 54).

(1) Identify the TSS of a *C. elegans* target gene, considering the phenomenon of *trans*-splicing that might obscure relevant TSSs (39). If applicable, give preference to the representative adult TSS over the representative embryonic TSS. (2) Designate −50 to −400 bp upstream of the selected TSS as the region from which to select all sgRNA target sequences (44). (3) From this region, select appropriate 20 nt sgRNA target sequences flanked by an NGG protospacer-adjacent motif based on established computational design rules predicting their on- and off-target scores (53, 54). Preferably select sgRNA target sequences with on- and off-target scores >50 and give preference to those with the highest possible scores. Note that orientation of sgRNA target sequences relative to the TSS, that is, whether they are located on the same or on opposite DNA strands, appears negligible and should not be used as a criterion for exclusion of otherwise suitable sgRNA target sequences (25).

All sgRNA target sequences used for proof-of-principle experiments in this publication were designed strictly according to these rules and are contained in Table S2. Specifically, we designed sgRNA target sequences for the promoters of the genes *hif-1*/F38A6.3 (WBGene00001851) and *hsf-1*/Y53C10A.12 (WBGene00002004). Additionally, sgRNA SCR sequences were designed using random 20 nt sequences with a GC content of 50% that were confirmed by BLAST to not have any significant matches with the known *C. elegans* genome.

For large-scale prediction of promoter-specific sgRNA target sequences, we focused on all *C. elegans* genes with experimentally confirmed representative embryonic and/or adult TSSs (39), a total of more than 13,000 genes representing approximately 65% of all known *C. elegans* genes. The rules described previously were used for batch computational prediction using Ensembl BioMart release 97 (55), the guide RNA selection tool CRISPOR (56), and custom JavaScript, Perl, and Python scripts. Table S3 contains all relevant information together with the predicted sgRNA target sequences, ranked by their on-target efficiency scores according to the method by Doench *et al.* (54). Each individual sgRNA target sequence is given in the format XX_NNNNNNNNNNNNNNNNNNNN, where XX is its on-target efficiency score and N a nucleotide.

### Cloning procedures

*C. elegans* dCas9::VP64 overexpression vector: We designed a DNA fragment flanked by attB1 and attB2 recombination sites and containing a Cas9 coding sequence optimized for efficient expression in *C. elegans* (based on Addgene plasmid #47549) (57). This sequence was altered by introducing two amino acid mutations (D10A and H840A) known to inactivate the endonuclease function of Cas9 (resulting in dCas9) (14). At the 5′ end of the dCas9 sequence, a sequence encoding a 3xFLAG-tag and a SV40 nuclear localization signal (NLS) and at the 3′ end, a sequence encoding another SV40 NLS, a VP64 transactivation domain, and an HA-tag (based on Addgene plasmid #47107) (26) followed by a stop codon was added. The full sequence (attb1_3xFLAG::SV40-NLS::dCas9::SV40-NLS::VP64::HA_attB2) was obtained using a custom DNA synthesis service and inserted into the Gateway pDONR221 vector (Thermo Fisher Scientific; catalog no.: #12536017) using recombination as mediated by the Gateway BP Clonase II Enzyme Mix (Thermo Fisher Scientific; catalog no.: #11789020), resulting in the vector pENTRY_dCas9-VP64. A vector containing 2300 bp of the *C. elegans sur-5*/K03A1.5 (WBGene00006351) promoter was generated by amplifying the promoter sequence from genomic DNA using primers, introducing attB4 and attB1R recombination sites at the 5′ and 3′ end, respectively. The PCR product was inserted into the Gateway pDONRP4-1R vector (Thermo Fisher Scientific; catalog no.: #12536017) as aforementioned, resulting in the vector pENTRY_sur5p. The newly generated vectors pENTRY_psur5 and pENTRY_dCas9-VP64 were inserted into the destination vector pdestMB14 (Addgene; plasmid #26415) (58) using the Gateway LR Clonase II Enzyme Mix (Thermo Fisher Scientific; catalog no.: #11791020), to obtain vector pdestMB14_sur5p-dCas9-VP64 (deposited at and available from Addgene with the ID 177788).

L4440-derived sgRNA expression vector: By using the standard 2790 bp L4440 RNAi empty vector (Addgene; plasmid #1654) as a template for mutagenesis PCR with the Q5 Site-Directed Mutagenesis Kit (New England Biolabs; catalog no.: #E0554S), the intermediate vector L4440-BioBrick was generated. In this 2585 bp vector, nucleotides 1982 to 2204 of the original L4440 vector, including the bidirectional T7 promoters, were deleted and replaced with an 18 nt sequence (5′GAATTCAAGCTTCTGCAG) that contains EcoRI, HindIII, and PstI restriction sites. The EcoRI and PstI restriction sites are positioned in such a way as to conform to the BioBrick assembly standard (47). In addition, a BsaI restriction site in the backbone of L4440 was destroyed. Into L4440-BioBrick, a 329 bp sequence containing two sgRNA expression cassettes (see Fig. S1*A*) was inserted *via* EcoRI and PstI restriction sites to obtain L4440_BioBrick-sgRNA (deposited at and available from Addgene with the ID 177783). Individual promoter-specific sgRNA target sequences or SCR were inserted into L4440_BioBrick-sgRNA *via* the BbsI and BsaI restriction sites and using oligos with appropriate overhangs. See Table S2 for vector designations and Addgene IDs, where all of these are available from.

### Bombardment and generation of stable *C. elegans* strains

The pdestMB14_sur5p-dCas9-VP64 vector (Addgene ID 177788) was transformed into the *unc-119*-deficient *C. elegans* strain HT1593 by microparticle bombardment using the biolistic particle delivery system PDS-1000/He (Bio-Rad) according to the manufacturer’s instructions and previously described protocols (59). For identification and genotyping of dCas9::VP64–positive nematodes, we conducted single-nematode PCR using the primers Ce_dC9V_gt_fwd (5′-GAGGACAACGAGCAAAAGCA-3′) and Ce_dC9V_gt_rev (5′-GAGGTTGGTGAGGGTGAAGA-3′). We obtained a stable insertion of the construct into the genome, as confirmed by PCR-based offspring analysis over several generations. We also verified the presence of the recombinant protein by immunoblotting and immunofluorescence, as detailed later. The resulting strain was called dCas9::VP64 and outcrossed a total of four times against WT N2. Unless explicitly stated otherwise, we used this four-time outcrossed dCas9::VP64 strain for all experiments reported in this publication. This strain (MIR249) is available from the CGC.

### *C. elegans* lifespan assays

All *C. elegans* lifespan assays were performed at 20 °C according to standard protocols as previously described, explicitly omitting FUdR (60). Briefly, adult nematodes were allowed to lay eggs for 4 to 9 h, and the resulting eggs were incubated for 64 h at 20 °C on NGM agar plates inoculated with OP50 to obtain a synchronized population of young adult nematodes. For a typical lifespan assay, 100 young adult nematodes per condition were manually transferred to NGM agar plates (30–35 nematodes per 55 mm Petri dish, supplemented with ampicillin and IPTG as described previously for all experiments using HT115 bacteria) inoculated with the respective bacteria as indicated. For the first 10 to 12 days, nematodes were transferred daily and afterward every 2 to 3 days. Nematodes showing no reaction to gentle stimulation were scored as dead. Nematodes that crawled off the plates, displayed internal hatching or a protruding vulva were censored. All key lifespan assays were repeated by different individual researchers in two independent laboratories.

### Protein extraction from *C. elegans*

Per sample, a mixed population of nonstarved nematodes was collected from a 90 mm NGM agar plate by washing with 10 ml S buffer and transferred to a 15 ml reaction tube. Samples were centrifuged for 1 min at 1300*g*, supernatants were discarded to 0.5 ml, and the nematodes were transferred to 1.5 ml reaction tubes. The remaining supernatant of each sample was carefully discarded after centrifugation for 1 min at 20,000*g*. For extraction of total protein, approximately 100 μl radioimmunoprecipitation assay buffer (equal to twice the nematode pellet volume), containing 1× Halt Protease and Phosphatase Inhibitor Cocktail (100×) (Thermo Fisher Scientific; catalog no.: #78440), was added per sample. Nematodes were cracked by three cycles of freeze-thawing (freeze samples for 10–20 s in liquid nitrogen, incubate in RT water bath for 2–3 min until samples begin to thaw) and sonication on ice (20 s at 80% amplitude). Samples were then centrifuged for 10 min at 12,000*g* and 4 °C, and the supernatants containing the extracted total proteins were transferred to new reaction tubes. For protein quantification, the “Roti-Nanoquant” (Carl Roth; catalog no.: #K880) reagent, along with bovine serum albumin standard, was used according to the manufacturer’s instructions. Samples were then either used directly for immunoblotting or stored at −80 °C until further use.

### Immunoblotting

Per sample, 40 μg of *C. elegans* total protein extract was boiled for 10 min at 95 °C in 1× Laemmli sample buffer. The samples were then used for a standard SDS-PAGE in a Mini-PROTEAN Tetra Cell (Bio-Rad Laboratories) electrophoresis chamber according to the manufacturer’s instructions. Following electrophoresis, transfer of the proteins to a polyvinylidene fluoride membrane was achieved using the Mini Trans-Blot Cell (Bio-Rad Laboratories) blotting module. After blotting, the membrane was blocked for 30 min in Tris-buffered saline with Tween-20 with 5% nonfat dry milk and then incubated overnight at 4 °C with the ANTI-FLAG M2 antibody (Sigma–Aldrich; catalog no.: #F3165) at a dilution of 5 μg/ml. Incubation with the horseradish peroxidase–linked secondary antimouse antibody (Cell Signaling Technology; catalog no.: #7076) was performed for 1 h at RT and a dilution of 1:1000. The Clarity Western ECL Substrate (Bio-Rad Laboratories; catalog no.: #1705060) was used for chemiluminescent immunoblot detection and a ChemiDoc Imaging System (Bio-Rad Laboratories) for documentation.

### RNA extraction from *C. elegans*

Per sample, a 90 mm NGM agar plate containing 100 μg/ml ampicillin and 1 mM IPTG was inoculated with 500 μl of 2× concentrated *E. coli* HT115 carrying the desired L4440_BioBrick-sgRNA vector. Following 24 h preincubation of the inoculated plates, ca. 200 synchronized young adult nematodes were transferred onto each plate. Using S buffer, nematodes were transferred daily onto new plates that were inoculated and preincubated as before. After 48 to 72 h incubation at 20 °C, nematodes were collected by washing with 10 ml ice-cold S buffer and transferred to prechilled 15 ml reaction tubes on ice. Samples were then centrifuged for 1 min at 1300*g* and 4 °C, supernatants were discarded to 0.5 ml, and the nematodes were transferred to prechilled 1.5 ml reaction tubes on ice. The remaining supernatant of each sample was carefully discarded after centrifugation for 1 min at 20,000*g* and 4 °C, and the resulting nematode pellets were immediately flash-frozen in liquid nitrogen and stored at −80 °C until RNA extraction was performed.

For extraction of total RNA, 500 μl of TRIzol Reagent (Thermo Fisher Scientific; catalog no.: #15596018) was added to each frozen nematode pellet. Nematodes were cracked by five cycles of freeze-thawing (freeze samples for 20 s in liquid nitrogen and incubate at 37 °C under constant shaking for 2–3 min until samples begin to thaw) and afterward incubated for 5 min at RT. After the addition of 200 μl chloroform per sample and vigorous shaking for 15 s, samples were incubated for 3 min at RT and then centrifuged for 20 min at 12,000*g* and 4 °C. The upper aqueous phase (200–300 μl) of each sample was transferred to a new 1.5 ml reaction tube, mixed with 1.1× volume of isopropyl alcohol and 0.16× volume of 2 M NaAc pH 4.0, and incubated for 10 min at RT. Nucleic acids were pelleted by centrifugation for 20 min at 12,000*g* and 4 °C, and supernatants were discarded. Nucleic acid pellets were washed twice with 1 ml of 80% ethanol and collected by centrifugation for 10 min at 7500*g* and 4 °C. After complete removal of ethanol and air-drying for 5 to 10 min, each pellet was dissolved in 50 μl of nuclease-free H2O. The concentration of the resulting RNA was measured using an LVis Plate on a CLARIOstar microplate reader (BMG LABTECH), and RNA-integrity was checked by agarose gel electrophoresis. RNA samples were either used directly for reverse transcription or stored at −80 °C until further use.

### Reverse transcription-quantitative PCR

Reverse transcription of RNA to cDNA was performed using the High-Capacity cDNA Reverse Transcription Kit (Thermo Fisher Scientific; catalog no.: #4368813) with the supplied RT Random Primers according to the manufacturer's instructions. Quantitative real-time PCR was carried out on a ViiA 7 Real-Time PCR System (Thermo Fisher Scientific) using the SYBR Select Master Mix (Thermo Fisher Scientific; catalog no.: #4472919) in 384-well plates according to the manufacturer’s instructions. In a typical reaction, final concentrations of 1 ng/μl cDNA template and 200 nM forward and reverse primer were used in a total reaction volume of 10 μl per well. Two well-established *C. elegans* reference genes, namely *cdc*-*42*/R07G3.1 (WBGene00000390) and *pmp*-*3*/C54G10.3 (WBGene00004060) (61), were used for normalization. Amplification of *hif-1* was performed with primers F38A6.3_hif1_fwd (5′-GCCACAATTTGTCGACTGCG-3′) and F38A6.3_hif1_rev (5′-CTCGACCTGTTAAATCTGTCTGTG-3′) and of *hsf-1* with primers Y53C10A.12_hsf1_fwd (5′-GTAATGGCAGAGATGCGTGC-3′) and Y53C10A.12_hsf1_rev (5′-TCCAGCACACCTCGTTTCG-3′). Quantification cycles (Cq) of target and reference genes were determined using the QuantStudio Real-Time PCR Software v1.3 (Thermo Fisher Scientific) according to the method described in the associated user guide (Thermo Fisher Scientific, Publication #4489822). Normalized fold expression of target genes was calculated following a data workup procedure yielding results equivalent to the ΔΔCq method (62).

### Next-generation sequencing (RNA-Seq)

To identify genes regulated in MIR249 (dCas9::VP64) *versus* WT N2 nematodes raised on *E. coli* HT115 SCR bacteria, RNA-Seq and data analysis were performed using three independent biological samples of total RNA extracted for each condition, as previously described (49). Samples were obtained from synchronized populations at 48 h postdevelopment.

#### Library preparation

The quality of the isolated RNA was determined with a Qubit (1.0) Fluorometer (Life Technologies) and a Bioanalyzer 2100 (Agilent). Only those samples with a 260 nm/280 nm ratio between 1.8 to 2.1 and a 28S/18S ratio within 1.5 to 2 were further processed. The TruSeq RNA Sample Prep Kit v2 (Illumina; #RS-122-2001/2) was used in the succeeding steps. Briefly, total RNA samples (100–1000 ng) were poly A enriched and then reverse transcribed into double-stranded cDNA. The cDNA samples were fragmented, end-repaired, and polyadenylated before ligation of TruSeq adapters containing the index for multiplexing. Fragments containing TruSeq adapters on both ends were selectively enriched with PCR. The quality and quantity of the enriched libraries were validated using Qubit (1.0) Fluorometer and the Caliper GX LabChip GX (Caliper Life Sciences). Products are a smear with an average fragment size of approximately 260 bp. The libraries were normalized to 10 nM in Tris-Cl 10 mM, pH 8.5 with 0.1% Tween 20.

#### RNA-Seq

RNA-Seq was performed in one multiplex on the Illumina Novaseq 6000 single-end at 100 bp, with a sequencing depth of 25 million reads per sample.

#### RNA-Seq data analysis

Bioinformatic analysis was performed within the data analysis framework SUSHI (63). Quality controlled reads (adapter trimmed with fastp: options “--trim_front1 1 —trim_tail 1 --cut_tail 20 --trim_poly_x --poly_x_min_len 10 --length_required) were aligned to the *C. elegans* reference genome (Ensembl WBcel235 [https://www.ncbi.nlm.nih.gov/assembly/GCF_000002985.6]) using the STAR aligner (64). Expression counts were computed using feature Counts in the Bioconductor package Subread (65). Differential expression analysis was performed using edgeR (66). To determine differently regulated genes, a fold-change cutoff ≥2 and an FDR cutoff of <0.01 were applied. The corresponding datasets generated for this study can be found in the NCBI’s Gene Expression Omnibus, GEO Series accession number GSE202213 (https://www.ncbi.nlm.nih.gov/geo/query/acc.cgi?acc=GSE202213).

### Fluorescence microscopy

Strain MIR249 was intercrossed with strain TJ375 (gpIs1 [*hsp-16.2*p::GFP]), in which expression of GFP is controlled by the *hsp-16.2* promoter. The resulting strain was confirmed for presence of the dCas9::VP64 construct by genotyping PCR and for *hsp-16.2*p-driven expression of GFP under the fluorescence microscope. Strains were maintained on OP50 bacteria at 20 °C. Gravid adults were treated with sodium hypochlorite solution to obtain eggs. The eggs were grown on HT115 SCR or HT115 sgRNA *hif-1* A or B bacteria until young adult. At young adult stage, the plates were heat-shocked at 33 °C for 2 h. After recovery from shock for 6 h at 20 °C, approximately 30 animals were mounted onto 2% agarose pads and anesthetized with 20 mM levamisole for imaging. Animals were captured at 10× magnification with one or two field of view and then stitched together afterward using ImageJ (https://imagej.nih.gov/ij/). Total fluorescence intensity was quantified by running a python script, GreenIntensityCalculator (available at https://github.com/Ewaldlab-LSD/GreenIntensityCalculator) in ImageJ. Statistical analysis of the quantified data was performed using GraphPad Prism. The experiment was performed in four independent replicate experiments with fluorescence intensity of >120 animals quantified in total for each condition.

### Immunofluorescence

Worms were washed from one 10 cm plate using S-buffer and snap-frozen in liquid nitrogen on top of a poly-D-lysine-coated (Sigma; #P7405) glass slide. Afterward, they were fixed for 20 min at −20 °C in a methanol:acetone 1:1 ratio solution. They were then rinsed twice in PBS + 0.1% Tween-20 and blocked for 1 h with a solution of 1% bovine serum albumin, 10% goat serum in PBS + 0.1% Tween-20. Slides were then incubated with 200-fold diluted rat anti-HA tag antibody (Roche; #10744700) in blocking solution for 2 h at RT. They were then washed three times, 10 min each, with PBS + 0.1% Tween-20 and incubated for 45 min in 500-fold diluted secondary antirat antibody in blocking solution (Invitrogen; #A21247). After three additional 10 min washes in PBS + 0.1% Tween-20, coverslips were mounted using mounting media ProLong Diamond Antifade Mountant (Invitrogen; #P36966) and incubated overnight at RT prior to imaging

### Confocal imaging

Confocal imaging was performed using an Olympus FluoView 3000 (Olympus Corporation) microscope with inverted stand. Fluoview FV31S-SW software (https://www.olympus-lifescience.com/en/downloads/detail-iframe/?0[downloads][id]=847252002) was used for image acquisition. Single plane images of individual nematodes were acquired using an UPLFLN 20× objective (exc/em Alexa Fluor 647: 650/671).

### Statistical analyses

Statistical analyses for all data except those from lifespan assays were carried out using a *t* test with appropriate parameters, that is, a two-tailed unequal variances *t* test for comparison of the unpaired control *versus* treatment groups. For comparing distributions between different groups in the lifespan assays, statistical calculations were performed using JMP software version 9.0 (SAS Institute), applying the log-rank test. All other calculations were performed using Microsoft Excel or GraphPad Prism 8 (GraphPad Software). *p*-Values are reported in detail without the use of arbitrary star ratings.

## Data availability

All data supporting the findings of this study are available within this paper and its supporting information.

## Supporting information

This article contains supporting information.

## Conflict of interest

The authors declare that they have no conflicts of interest with the contents of this article.
