# Supplementary material for: Ingestion of single guide RNAs induces gene overexpression and extends lifespan in Caenorhabditis elegans via CRISPR activation
Source: J Biol Chem. 2022 May 27;298(7):102085. doi: 10.1016/j.jbc.2022.102085 (PMC9243178; doi:10.1016/j.jbc.2022.102085)
Supplement: Supplemental Figure S1 [file mmc4.pdf]

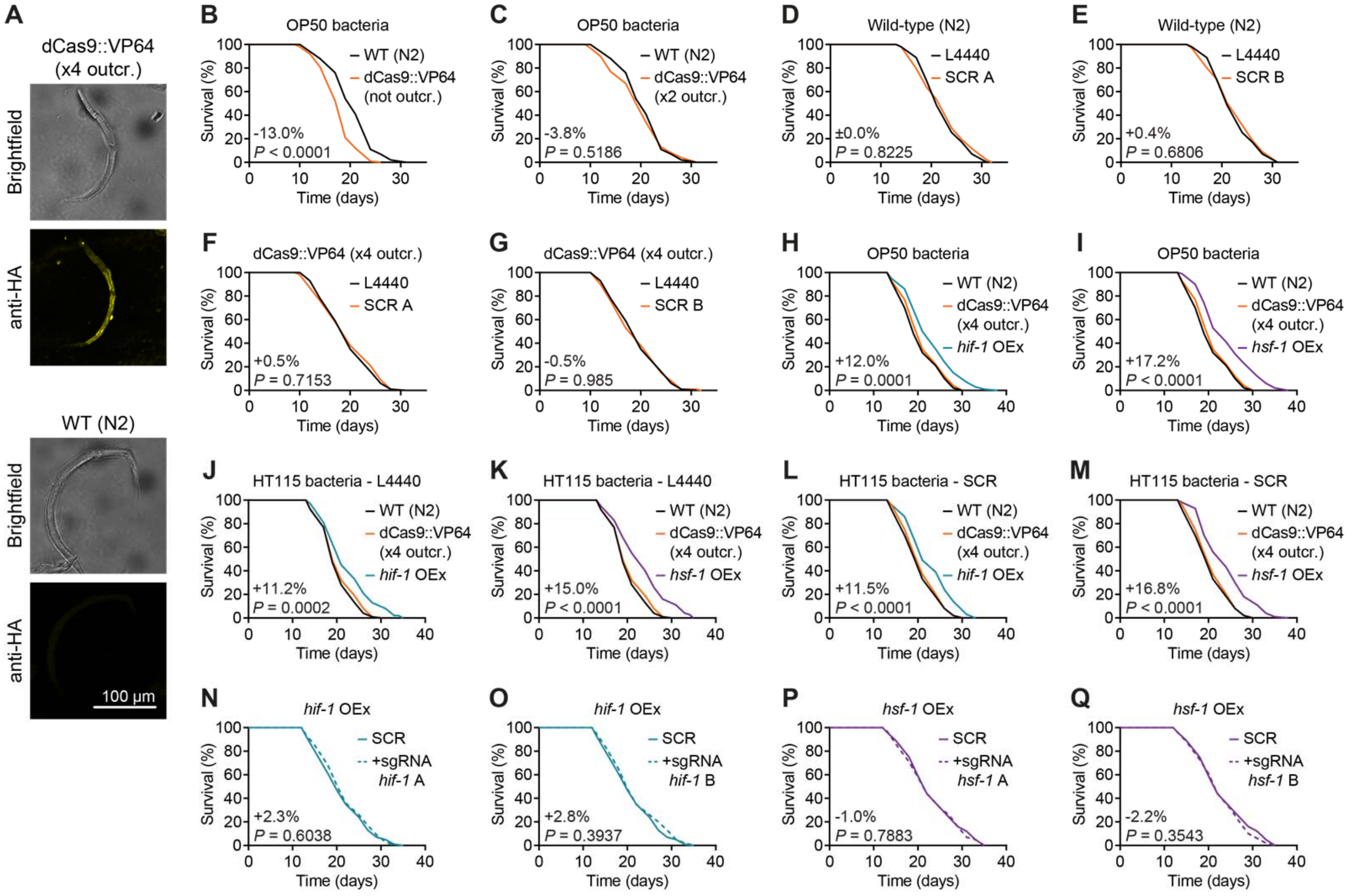

Figure S1. Additional control experiments for CRISPR activation in *C. elegans* by bacterial delivery of sgRNAs.

A, Representative images of immunofluorescence confocal microscopy with dCas9::VP64 (upper panels) or wild-type (WT) N2 nematodes (lower dpanels) and using an anti-HA antibody to detect HA-tagged dCas9::VP64. *B-C*, Lifespan assay on OP50 bacteria of WT nematodes vs Cas9::VP64 either not outcrossed (*B*) or outcrossed twice (*C*) against WT. *D-E*, Lifespan assay of WT nematodes on HT115 bacteria carrying the L4440 empty vector or the L4440_BioBrick-sgRNA vector expressing sgRNA scramble (SCR) control sequences set A (*D*) or set B (*E*). *F-G*, Lifespan assays using the same bacteria as in (*D-E*) and four times-outcrossed dCas9::VP64 nematodes. *H-M*, Lifespan assays on the indicated bacteria (OP50, HT115 L4440, HT115 SCR) and using *hif-1* (ZG580) or *hsf-1* (CF1824) overexpression (OEx) strains vs WT and dCas9::VP64 nematodes in each case. Percentage changes in lifespan and *P*-values correspond to the respective comparisons of *hif-1* or *hsf-1* OEx vs WT in each assay. *N-Q*, Lifespan assays of *hif-1* or *hsf-1* OEx on HT115 SCR bacteria vs the indicated HT115 sgRNA bacteria (*hif-1* A or B, *hsf-1* A or B). *P*-values of *C. elegans* lifespan assays were determined by log-rank test. See Table S1 for detailed lifespan assay statistics.
